# Echocardiography protocol for early detection of cardiac dysfunction in childhood cancer survivors in the multicenter DCCSS LATER 2 CARD study: Design, feasibility, and reproducibility

**DOI:** 10.1111/echo.15081

**Published:** 2021-05-20

**Authors:** Remy Merkx, Jan M. Leerink, Elisabeth (Lieke) A.M. Feijen, Leontien C.M. Kremer, Esmée C. de Baat, Louise Bellersen, Elvira C. van Dalen, Eline van Dulmen‐den Broeder, Margriet van der Heiden‐van der Loo, Marry M. van den Heuvel‐Eibrink, Chris L. de Korte, Jacqueline Loonen, Marloes Louwerens, Angela H.E.M. Maas, Yigal M. Pinto, Cécile M. Ronckers, Arco J. Teske, Wim J.E. Tissing, Andrica C.H. de Vries, Annelies M.C. Mavinkurve‐Groothuis, Helena J.H. van der Pal, Gert Weijers, Wouter E.M. Kok, Livia Kapusta, The Dutch LATER Study Group

**Affiliations:** ^1^ Department of Medical Imaging/Radiology Medical UltraSound Imaging Centre Radboud university medical center Nijmegen The Netherlands; ^2^ Department of Clinical and Experimental Cardiology Amsterdam UMC University of Amsterdam Amsterdam The Netherlands; ^3^ Princess Máxima Center for Pediatric Oncology Utrecht The Netherlands; ^4^ Department of Cardiology Radboud university medical center Nijmegen The Netherlands; ^5^ Department of Pediatric Oncology Amsterdam UMC VU University Amsterdam The Netherlands; ^6^ Trial‐ and Data Center Princess Máxima Center for Pediatric Oncology Utrecht The Netherlands; ^7^ Department of Hematology Radboud university medical center Nijmegen The Netherlands; ^8^ Department of Internal Medicine Leiden University Medical Center Leiden The Netherlands; ^9^ Department of Cardiology University Medical Center Utrecht Utrecht The Netherlands; ^10^ Department of Pediatric Oncology Beatrix Children's Hospital University Medical Center Groningen Groningen The Netherlands; ^11^ Department of Pediatrics Pediatric Cardiology Unit Tel Aviv Sourasky Medical Center Sackler School of Medicine Tel Aviv University Tel Aviv Israel; ^12^ Department of Pediatric Cardiology Amalia Children’s Hospital Radboud University Medical Center Nijmegen The Netherlands

**Keywords:** 2D echocardiography, cardiac toxicity, diastolic function, myocardial strain, systolic function

## Abstract

**Background:**

Cardiotoxicity is a well‐known side effect after anthracyclines and chest radiotherapy in childhood cancer survivors (CCS). The DCCSS LATER 2 CARD (cardiology) study includes evaluation of echocardiographic measurements for early identification of CCS at highest risk of developing heart failure. This paper describes the design, feasibility, and reproducibility of the echocardiography protocol.

**Methods:**

Echocardiograms from CCS and sibling controls were prospectively obtained at the participating centers and centrally analyzed. We describe the image acquisition, measurement protocol, and software‐specific considerations for myocardial strain analyses. We report the feasibility of the primary outcomes of systolic and diastolic function, as well as reproducibility analyses in 30 subjects.

**Results:**

We obtained 1,679 echocardiograms. Biplane ejection fraction (LVEF) measurement was feasible in 91% and 96% of CCS and siblings, respectively, global longitudinal strain (GLS) in 80% and 91%, global circumferential strain (GCS) in 86% and 89%, and ≥2 diastolic function parameters in 99% and 100%, right ventricle free wall strain (RVFWS) in 57% and 65%, and left atrial reservoir strain (LASr) in 72% and 79%. Intra‐class correlation coefficients for inter‐observer variability were 0.85 for LVEF, 0.76 for GLS, 0.70 for GCS, 0.89 for RVFWS and 0.89 for LASr. Intra‐class correlation coefficients for intra‐observer variability were 0.87 for LVEF, 0.82 for GLS, 0.82 for GCS, 0.85 for RVFWS and 0.79 for LASr.

**Conclusion:**

The DCCSS LATER 2 CARD study includes a protocolized echocardiogram, with feasible and reproducible primary outcome measurements. This ensures high‐quality outcome data for prevalence estimates and for reliable comparison of cardiac function parameters.

## 1 INTRODUCTION

Treatment of childhood cancer has drastically improved over the last decades, resulting in a 5‐year survival of over 80%, nowadays.^1^ However, as the population of long‐term childhood cancer survivors (CCS) increases, awareness has risen concerning their risk of various late treatment effects.^2^ Cardiotoxicity is a well‐known side effect of anthracyclines and radiotherapy involving the heart region and is responsible for substantial morbidity and mortality, even decades after therapy.^3^, ^4^ Besides coronary artery disease, valvular disease, pericardial disease, and arrhythmias,^5^ the most important manifestation of cardiotoxicity is cardiomyopathy, which can range from asymptomatic left ventricular dysfunction to overt or even fatal clinical heart failure. Surveillance guidelines recommend periodical echocardiographic examination for early detection of left ventricular dysfunction, with surveillance intervals based on cardiotoxic therapy exposures.^6^

More detailed risk stratification through sensitive detection tools may enable late‐effects clinicians to better prevent clinical heart failure in case of increased risk, or reduce surveillance burden in case of low risk. The most frequently reported systolic function parameter, left ventricular (LV) ejection fraction (LVEF), is considered the standard echocardiographic measure of LV dysfunction but is a rather late marker compared with measurements of myocardial strain.^7^, ^8^ Compared with magnetic resonance or nuclear imaging, echocardiographic LVEF comes with some disadvantage of a test‐retest variability of 5%‐10%‐points in expert echo‐laboratories, depending on a biplane or 3D approach used.^9^, ^10^, ^11^ In the last decade, effort has been put into refinement of risk estimation for the development of heart failure after cancer therapies, with little success for blood biomarkers but with a better outlook for more sensitive, early echocardiographic parameters.^12^, ^13^, ^14^ In other populations with (risk of) cardiovascular disease, global longitudinal strain (GLS) shows very promising results for earlier identification of those at risk for heart failure and death.^7^, ^8^, ^15^ In CCS, abnormal GLS is highly prevalent,^12^, ^16^ but its prognostic value has yet to be shown. Myocardial strain measurement has not been widely adopted in clinic, possibly hampered by the different algorithms used by different software vendors, which can be partly resolved by the use of vendor‐independent software.^17^

The Dutch Childhood Cancer Survivor Study (DCCSS) LATER cohort (1963‐2001) part 2; clinical visit and questionnaire study provides a unique opportunity to investigate cardiotoxicity, next to various late effects in a large sample of very long‐term survivors (DCCSS LATER 2 CARD study). This substudy includes solitary and combined analyses of echocardiography, electrocardiograms, and blood biomarkers.^18^ The echocardiographic measurements serve as primary study outcome and as reference for the biomarker and ECG studies. Specific aims of the echocardiography study are to evaluate the prevalence and associated (treatment and lifestyle related) risk factors of subclinical cardiac dysfunction in CCS compared with sibling controls, and with identify more sensitive echocardiographic markers of subclinical cardiac dysfunction.

As myocardial strain analysis is very comprehensive and results are influenced by the software and definitions used, it is of key importance to describe the methods in detail. Furthermore, the feasibility of (strain) measurements should be described, as they depend on image quality. Last, the measurements should be reproducible in research and clinical follow‐up. The overall design of the DCCSS LATER 2 CARD study has recently been published.^18^ Here, we describe the image acquisition, the protocol for offline conventional and strain measurements, as well as their feasibility and reproducibility, as part of the multicenter echocardiography study in the DCCSS LATER 2 CARD cohort.

## 2 METHODS

### 2.1 Patient population

The cross‐sectional DCCSS LATER study part 2 investigates late treatment effects in a nationwide cohort of 5‐year CCS, treated under the age of 18 years between 01‐01‐1963 and 31‐12‐2001.^18^ This baseline cohort comprises 6,165 CCS, of which 5,455 were alive at study inception. From this cohort, the DCCSS LATER 2 CARD study aimed to include 1,900 CCS for cardiac evaluation with echocardiography, electrocardiography, and blood biomarkers. Of these, 1,600 CCS were defined as risk group 1, who received well‐known cardiotoxic therapy (anthracyclines, mitoxantrone, radiotherapy on the heart region, solitary, or combined).^3^ Risk groups 2, 3, and 4 were study groups of at most 100 subjects each, having received either cyclophosphamide, ifosfamide, or vincristine, respectively, without any other studied treatment. A control group of untreated siblings was recruited to account for background cardiovascular risk. Subjects were recruited from 7 Dutch pediatric oncology centers, between February, 2016 and February, 2020. For risk group 1, a protocolled echocardiography was part of standard surveillance,^6^ whereas for the other risk groups and sibling controls, all diagnostics were obtained for research. For participants from risk group 1 who did not have an indication for a new surveillance echocardiogram during the study period (surveillance echocardiography was recently performed, or the participant was already under the care of a cardiologist), we obtained their most recent echocardiogram if performed no earlier than January 1, 2016. All participants gave their informed consent for the use of study, and clinical data and the medical ethic boards of all participating centers approved the study protocol.

### 2.2 Echocardiography

Pediatric and adult cardiologists developed the echocardiography protocol, in collaboration with the DCCSS LATER 2 CARD steering committee and the Dutch childhood cancer cardiac consortium.

#### 2.2.1 Image acquisition

Experienced sonographers acquired images on the locally available Philips (8%) or GE (92%) stations. Table 1 summarizes the requested images. From approximately halfway the inclusion period, we also included the right ventricle (RV) focused apical four‐chamber view in our protocol. For all images, three heart cycles were recorded, with these exceptions: five cycles for patients in atrial fibrillation, five cycles for tissue Doppler imaging (pulsed wave frame rate >180/s), and five cycles for strain analysis (preferred frame rate 60‐100/s and ratio with heart rate ≥3:4; minimum 45/s).^19^, ^20^ For color Doppler, Nyquist limit was set at 50‐70 cm/s.

**TABLE 1:** Required images in DCCSS LATER 2 cardiology echocardiography protocol

	Clinical use (including measurements)^a^	Additional (images only)
**Left lateral decubitus position**		
Parasternal		
Long‐axis view	2D	2D: LVOT zoom
	M‐mode: LV and LA/Ao	
	Color Doppler: MV and AoV	
Short‐axis view	2D: apical, mid‐ventricular, MV, AoV level	2D: 5 cycles mid‐ventricular^b^
	Color Doppler: MV, AoV, PV, TV	
	CW: TV regurgitation	CW: PV outflow and regurgitation
Apical		
4‐chamber view	2D: atria and ventricles in 1 view^c^	2D: 5 cycles zoom LV; 5 cycles RV focused
	M‐mode: TAPSE, color MV inflow	M‐mode: MAPSE (lateral)
	Color Doppler: MV and TV	
	CW: TV regurgitation	
	PW: MV and right upper pulmonary vein inflow	PW: TV inflow
	PW‐TDI: LV septal and lateral basal wall^d^	PW‐TDI: RV lateral basal wall^d^
5‐chamber view	2D	
	Color Doppler: AoV and LVOT	
	CW: AoV outflow	CW: between AoV and MV for valve timing
	PW: LVOT	
2‐chamber view	2D: atrium and ventricle in 1 view^c^	2D: 5 cycles zoom LV^b^
	Color Doppler: MV	
3‐chamber view	2D: atrium and ventricle in 1 view^c^	2D: 5 cycles zoom LV^b^
	Color Doppler: MV and AoV	
**Supine position**		
Subcostal	2D: long‐axis view, IVC view	2D: 4‐chamber view
	M‐mode: IVC respiratory variation	
	Color Doppler: Hepatic vein if TR, abdominal Ao if AR	
	PW: Hepatic vein if TR, abdominal Ao if AR	
Suprasternal	Color Doppler: Ascending and descending Ao if AR	
	PW: Ascending and descending Ao if AR	

Abbreviations: Ao, aorta; AoV, aortic valve; AR, aortic regurgitation; CW, continuous wave Doppler; IVC, inferior vena cava; LA, left atrium; LV, left ventricle; LVOT, left ventricular outflow tract; MAPSE, mitral annular systolic plane excursion; MV, mitral valve; PV, pulmonary valve; RV, right ventricle; TAPSE, tricuspid annular systolic plane excursion; TDI, tissue Doppler imaging; TR, tricuspid regurgitation; TV, tricuspid valve.

^a^ Standard items for guideline‐based clinical evaluation in risk group 1, only images acquired in other risk groups.

^b^ 5 cycles, frame rate >60/s for strain measurements, recommended 75% of heart rate. Avoid sector size reductions.

^c^ Avoid foreshortening. Provide atrial‐focused images if no optimal alignment.

^d^ 5 cycles, PW Doppler frame rate >180/s, sector size reductions allowed.

#### 2.2.2 Data storage and handling

Images were locally stored and pseudonymized. Raw DICOM files were transferred to our echocardiography core laboratory at the Radboud university medical center, Nijmegen, via protected storage‐ or exchange media. Extraction of our offline measurement results from the analysis software was automated using custom scripts, and the data were then imported in a web‐based database (Castor EDC, Ciwit BV, The Netherlands).

#### 2.2.3 Measurement protocol

For reader convenience, we refer to conventional (all but strain) and strain measurements. Echocardiograms obtained for standard care were analyzed by the sonographer and an imaging cardiologist. For “research‐only” echocardiograms, only images (without online measurements) were stored. All measurements were (re)performed offline at the core laboratory by one out of two observers (RM or JL). The observers were blind for all participant information such as previous cancer diagnosis, therapy modalities and doses, cardiovascular risk factors, electrocardiographic findings, and blood tests. Analysis of conventional parameters for research‐only participants was performed upon receipt in the core laboratory, to be able to report unexpected findings (intra‐cardiac tumors, congenital heart disease, valve dysfunction, myocardial dysfunction) to their own physician within six months, as indicated by the ethical committee. Strain analyses were performed at a different time point, blinded from clinical information, and conventional echocardiographic measurements.

Conventional measurements were performed in EchoPac 202 (GE Vingmed, Norway) for GE acquired images, or TomTec Arena 2.31 (TomTec Imaging Systems GmbH, Germany) for images from Philips stations. Myocardial strain analyses were performed using the vendor‐independent TomTec 2D Cardiac Performance Analysis software v1.4. We expect minimal impact of the different image acquisition stations on the strain results, as >90% of the images were acquired on GE stations.^9^, ^21^ All offline measurements are shown in Table 2.

**TABLE 2:** Standard echocardiographic measurements and derived parameters

**Domain**		
(view)	Primary measurements	Calculated data
**LV structure and systolic function** ^a^		
PLAX^b^	LV end‐diastolic & ‐systolic diameter (LVEDD, LVESD)	Fractional shortening = (LVEDD‐LVESD)/LVEDD
	LV septal and posterior wall thickness (diastolic)	LV mass index (g/m^2^) = (0.832*[(IVS+LVID+PWT)^3^−LVID^3^]+0.6)/BSA
Apical 4‐chamber	Lateral MV annular plane excursion (MAPSE)	Corrected MAPSE =MAPSE / LV length
	Septal and lateral TDI s’ ^c^	
Apical 2 & 4‐chamber	LV end‐diastolic & ‐systolic volume (LVEDV, LVESV)	LVEF = (LVEDV ‐ LVESV)/LVESV
Apical 2, 3, 4‐chamber	Global longitudinal strain, strain rate	
PSAX (mid LV)	Global circumferential strain, strain rate	
**LV mechanical dispersion**		
Apical 2, 3, 4‐chamber	Longitudinal strain time‐to‐peak standard deviation	
**Diastolic function**		
Apical 4‐chamber	Mitral E‐ and A‐ wave velocity, E deceleration time	LV E/A ratio
	Mitral annular septal and lateral TDI e’ ^c^	LV E/e'ratio
	Pulmonary vein systolic (S) and diastolic (D) velocity	S/D ratio
	Tricuspid E‐ and A‐ wave velocity, E deceleration time	
**Valve structure and function** ^d^		
PLAX	LVOT diameter	AVA_VTI_ = (π*VTI_LVOT_* (LVOT diameter/2)^2^)/VTI_AoV_
PSAX (AoV level)	TR and PV Vmax	AVA_Vmax_ = (π*V_LVOT_* (LVOT diameter/2)^2^)/V_AoV_
Apical 3 or 5‐chamber	Vmax LVOT, Vmax AoV, VTI LVOT, VTI AoV	AVA index (cm/m^2^) = AVA / BSA
**LA structure and function**		
PLAX	LA diameter	
Apical 2 & 4‐chamber	LA end‐diastolic area & volume (LAEDV)	LA volume index (ml/m^2^) = LAEDV / BSA
Apical 4 chamber	LA reservoir, conduit & booster pump longitudinal strain	
**RV systolic function**		
Apical 4 chamber	Lateral TV annular plane excursion (TAPSE)	
	Lateral TDI s’ ^c^	
	RV and RV free wall longitudinal strain	
**Congestion signs**		
	Inferior vena cava diameter (in‐ and exspiration)	Respiratory variation (%)
**Timing**		
Apical 5‐chamber	Ejection time, IVRT, IVCT, R‐AvC	Ejection time (ET), IVRT, IVCT
		Tei index =ET / (IVCT +ET + IVRT)
Apical 2, 3, 4‐chamber	Time‐to‐peak strain (TTP)	TTP corrected =TTP in seconds / √ (cycle time in seconds))

Abbreviations: AoV, aortic valve; AR, aortic regurgitation; AVA, aortic valve area; BSA, body surface area; IVCT, isovolumic contraction time; IVRT, isovolumic relaxation time; LA, left atrium; LV, left ventricle; LVOT, left ventricular outflow tract; MR, mitral regurgitation; MS, mitral stenosis; MV, mitral valve; PLAX, parasternal long axis; PM, papillary muscle level; PSAX, parasternal short axis; PV, pulmonary valve; PW, Pulsed Wave Doppler; R‐AvC, aortic valve closure time; RV, right ventricle; TDI, Tissue Doppler imaging; TR, tricuspid regurgitation; Vmax, peak velocity; VTI, velocity time integral.

^a^ Besides visual inspection of global and regional wall motion.

^b^ 2D images are preferred over M‐mode.

^c^ TDI measurements are performed in three cardiac cycles.

^d^ Besides qualitative evaluation of morphology, valve opening, Color Doppler and additional measurements to determine severity.^38^, ^39^, ^40^

Observers subjectively rated the overall image quality (including acoustic window, artefacts, contrast) of an examination as “good,” “fair,’ “moderate,” or “poor,” allowing sensitivity analyses for the study results. When available, left ventricular dimensions were measured on 2D parasternal long‐axis images rather than M‐mode, to prevent oblique measurements.^22^ LVEF was calculated according to the biplane Simpson's method.^22^ EchoPac's speckle‐tracking based LVEF measurement (“autoEF”) was preferred to enhance reproducibility, though manual adjustment of endocardial contours, and reference frames remained possible. As not all participating centers routinely measured 3D LVEF, this measure was not included in the protocol. Tissue Doppler velocity measurements were performed and averaged over three regular cardiac cycles.

#### 2.2.4 Myocardial strain analysis

##### Event timing

The cardiac cycle with the best image quality was selected. For all cardiac chambers, end‐diastole was set at the QRS‐peak to pursue the highest reproducibility.^23^ End‐systole was set on the minimum of the volume or area curve derived by the software. Unless stated otherwise, we report end‐systolic strains for the LV and RV and peak reservoir strain for the left atrium (LA).^24^

##### Contour drawing and tracking

Endo‐ and epicardial 15‐point contours were manually applied to the LV wall in end‐systole, tracked by the software and, if necessary, manually adjusted in end‐diastole to cover the whole cardiac wall. Tracking quality was visually confirmed based on the superimposed grayscale images and the typical curve morphology. If tracking was inadequate, the contours were iteratively adjusted.^25^ In each view, the software divided the myocardium in six segments. Not‐tracked segments could be discarded to a total maximum of three segments in the three apical views and one segment in the short‐axis view.^23^ Per default, we report midwall (referred to by the software as myocardial) strain for the LV,^23^ which has been shown to be the least susceptible to potential foreshortening.^26^

Endocardial longitudinal strain of the RV was preferably measured from a RV focused apical four‐chamber view. To anchor the tracking of the RV apex, the interventricular septum was tracked, but then excluded to report the RV free wall longitudinal strain. Discarding of one of the three RV free wall segments was accepted when inadequately tracked. Endocardial longitudinal strain of the LA was measured from a non‐foreshortened apical four‐chamber view. As no segmentation applies to the LA, no segments could be discarded and global strain is calculated.^24^

##### Calculation

Left ventricular GLS was calculated as the mathematical average at end‐systole of the 18 segments derived from the three apical (four‐chamber, three‐chamber, two‐chamber) views,^27^ and not as the 16 segments‐ and ‘total line length”‐based GLS value derived by the TomTec software. This was necessary to allow the exclusion of not‐tracked segments from the GLS calculation. Furthermore, extraction of segmental values allows for calculation of individual wall strains (eg, septal, lateral, or right ventricular free wall). Our method might, however, overestimate the apical contribution to the contraction.^23^

**FIGURE 1:**
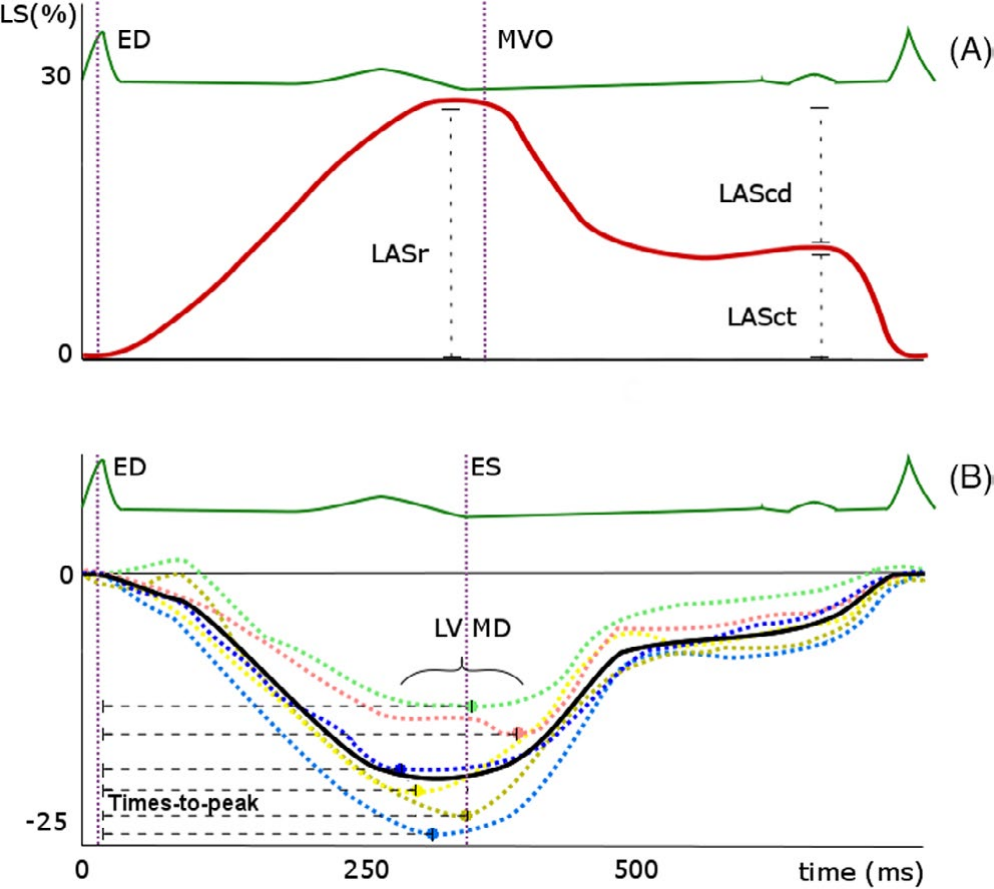
(A) Typical curve of left atrial (LA) longitudinal strain (LS), which has a positive value. End‐diastole (ED) is set at the QRS‐peak of the ECG, peak reservoir strain (LASr) typically precedes mitral valve opening (MVO). Passive (LAScd; conduit strain) and active (LASct; contractile strain) left atrial emptying are seen during ventricular diastole (B) Conceptualization of left ventricular mechanical dispersion (LV MD), which is the standard deviation of the 18 segmental time intervals from the QRS‐peak on the ECG to peak negative strain. Dashed curves are segmental longitudinal strain (LS) curves, the solid curve is the global longitudinal strain curve. ED =end‐diastole, ES =end‐systole (aortic valve closure, for which the surrogate of minimal volume is used)

Global circumferential strain (GCS) was measured in short‐axis view at the papillary muscle (mid‐ventricular) level and was averaged over six segments.^23^ RV free wall longitudinal strain was averaged over three free wall segments and for the LA, longitudinal reservoir strain was reported per default (no segmentation, Figure 1A).^24^ Strain measurements will be referenced as absolute values (ie, −21% is “better” than −18%).

Left ventricular mechanical dispersion, an important prognosticator for ventricular arrhythmias in multiple cardiac diseases,^28^ was defined as the standard deviation of the 18 segmental time intervals from the QRS‐peak to peak negative strain and expressed in msec (Figure 1B). Segmental strain curves that showed no (clear) negative peak (ie, dyskinetic and akinetic segments) cannot be included in assessment of mechanical dispersion, as they do not allow time‐to‐peak calculation.

### 2.3 Training and supervision

All sonographers on‐site were familiarized with the protocol. The two core‐laboratory observers were physicians and extensively trained and supervised by (pediatric) cardiologists with established experience in echocardiography and strain (LKa, WEK, LB). When observers doubted on endocardial border definition, quality of Doppler signals, severity of valvular dysfunction or presence of cardiac anomalies, studies were over‐read by LKa.

### 2.4 Study outcomes

Primary study endpoints were previously defined,^18^ and include prevalences of abnormal LVEF, GLS and diastolic function according to the current guidelines ^17^, ^22^, ^29^.

### 2.5 Statistical analysis

The feasibility of the primary outcomes of systolic and diastolic function and all strain analyses is presented as a percentage of all analyzed echocardiographic examinations. Differences in proportions between CCS and siblings were analyzed using Pearson chi‐square test. Core laboratory inter‐ and intra‐observer variability was tested in 30 randomly selected participants with sufficient image quality for the measurement concerned (RM, JL). Intra‐observer measurements were at least two weeks apart. The same cardiac cycle was used to exclude any temporal variability. Intra‐class correlation coefficients (ICC; two way mixed) and Bland‐Altman plots showing absolute and relative mean differences between observers and limits of agreement (calculated as mean −2SD and +2 SD) are presented as measures of agreement. The higher the ICC, the smaller the required sample size. With a probability of a type I error set at 5%, a sample of 30 patients provides a power of 91% to distinguish an ICC of 0.75 (good‐excellent) from 0.4 (poor), and 95% to distinguish and ICC of 0.9 from 0.7.^30^ Proportional bias (agreement depending on the actual measurement) was assessed by visual observation of Bland‐Altman plot and linear regression of the differences between observers on the averaged measured values. All statistics were performed in SPSS version 25 (IBM).

## 3 RESULTS

### 3.1 Patient population

**FIGURE 2:**
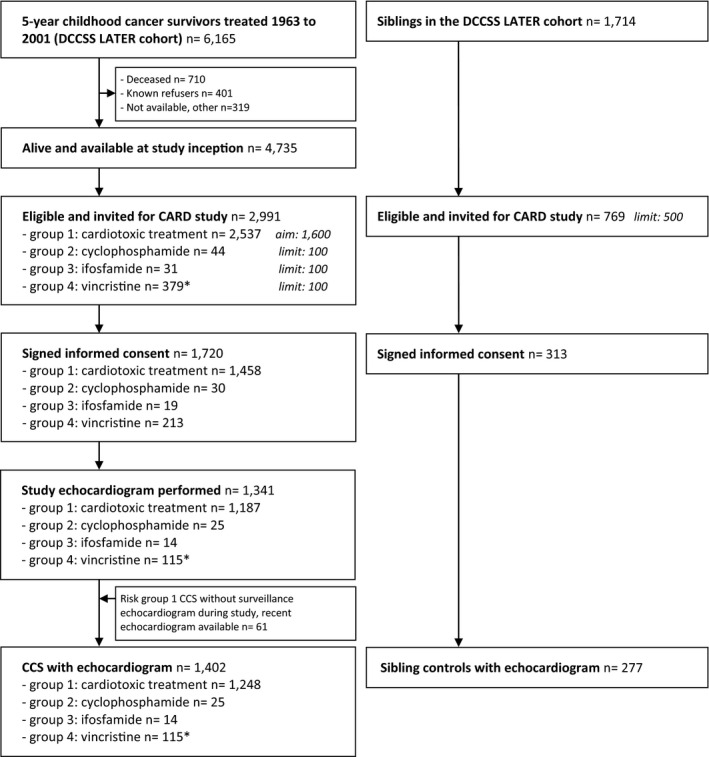
Inclusion flowchart of Dutch Childhood Cancer Survivors Study (DCCSS) LATER cardiology echocardiography study. CCS =childhood cancer survivor. * Study arm closed early after exceeding the predefined limit

As from the study closure date on March 1, 2020, protocolled echocardiograms of 1,618 participants were available in our core laboratory. Of these, 1,341 echocardiograms were obtained in CCS and 277 in sibling controls. We found 61 additional echocardiograms from CCS in risk group 1 to contain images compliant with our protocol, resulting in a total of 1,679 echocardiograms. A detailed inclusion flowchart is depicted in Figure 2.

### 3.2 Echocardiographic measurements

#### 3.2.1 Image quality and feasibility

Image quality was rated ‘good” in 606 (36%), ‘fair’ in 793 (47%), “moderate” in 245 (15%), and ‘poor’ in 35 (2%) of the participants. Image quality did not differ between CCS and siblings (‘good” quality in 36% vs 38%, ‘fair’ in 47% vs 47%, “moderate” in 15% vs 14%, and “poor’ in 2% vs 1%, respectively, *P*=.530). Feasibility of the primary outcome measurements and strain measurements is shown in Table 3. Except for diastolic function assessment (defined as ≥2 parameters available), all measurements were significantly more feasible in siblings than CCS. Biplane LVEF could be measured in 91% of CCS and 96% of siblings (*P* =.006), diastolic function in 99% and 100% (*P* =.10), respectively, and GLS in 80% and 91% (p = <0.001). All 18 segments were included in 75% of the GLS calculations in CCS and in 74% of those in siblings (*P*=.64).

**TABLE 3:** Feasibility of the primary echocardiographic measurements of cardiac function and strain measurements

Measure	Feasibility in CCS	Feasibility in siblings	*p*‐value
Biplane LV ejection fraction	91% (1276/1402)	96% (276/277)	0.006
Global longitudinal strain	80% (1126/1402)	91% (253/277)	<0.001
Global circumferential strain	86% (1212/1402)	89% (247/273)	0.21
Diastolic function ^a^	99% (1384/1402)	100% (277/277)	0.10
TAPSE	98% (1380/1402)	100% (277/277)	0.02
RV free wall strain	57% (805/1402)	65% (180/277)	0.02
LA reservoir strain	72% (1013/1402)	79% (218/277)	0.03

Note

Feasibility is shown as the % and n measurements possible out of all currently analyzed examinations: %, n/total.

Abbreviations: CCS, childhood cancer survivors; LA, left atrium; LV, left ventricle; RV, right ventricle​; TAPSE, tricuspid annular plane systolic excursion.

^a^ At least 2 parameters available out of: LA volume, tricuspid regurgitation jet, e’ or E/e’.^29^

Supplemental Figure 1 depicts the feasibility of biplane LVEF, TAPSE and GLS for different image qualities, as examples of a B‐mode, an M‐mode, and a strain measurement. In 18 (30%) of the 61 additional examinations, GLS could not be measured (of whom 15 had compressed images stored). A total of 81 examinations (all but two in CCS) were stored in compressed format.

#### 3.2.2 Core laboratory variability

**FIGURE 3:**
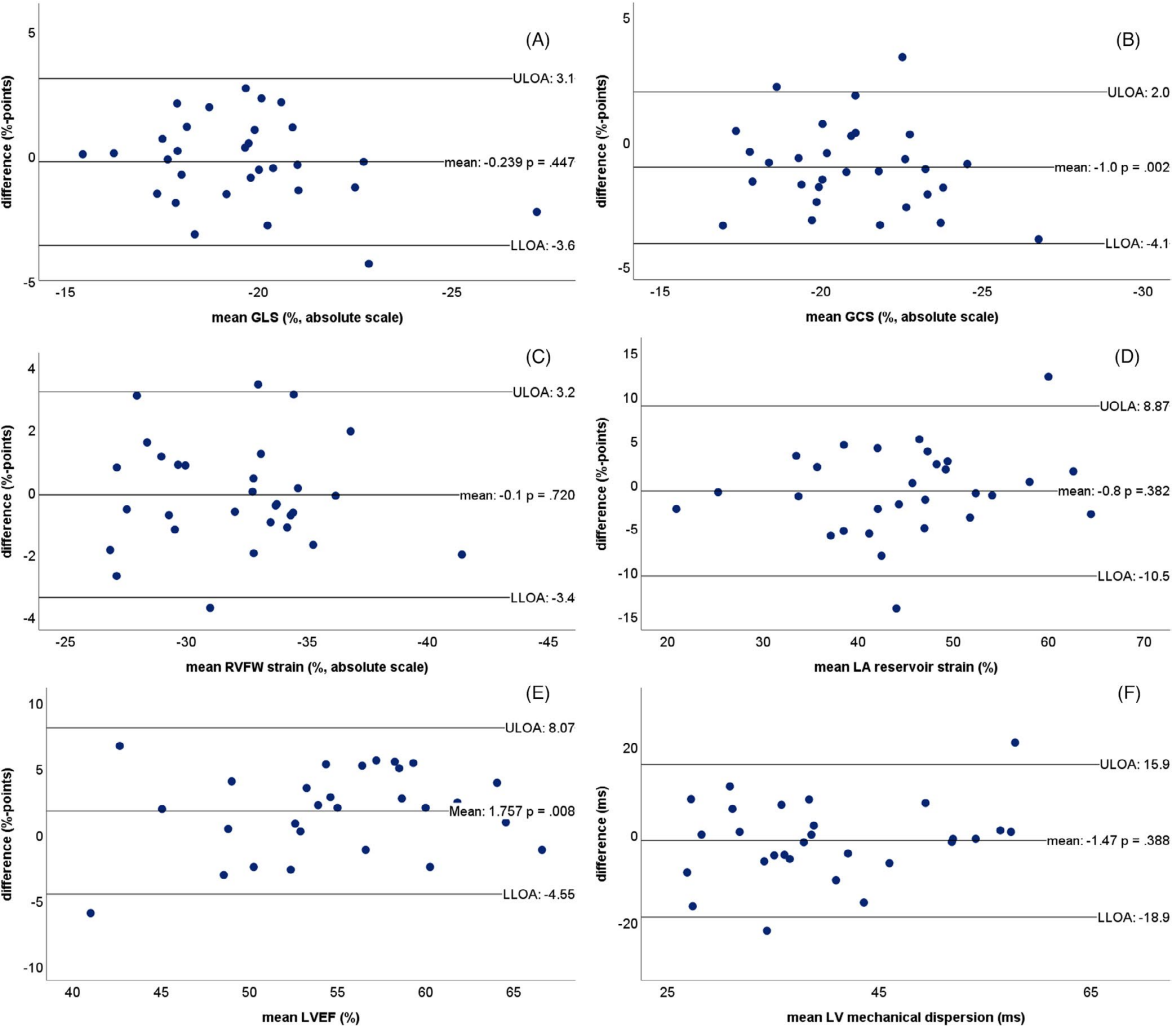
Bland‐Altman plots for inter‐observer variability for (A) global longitudinal strain (GLS), (B) global circumferential strain (GCS), (C) right ventricular free wall (RVFW) longitudinal strain, (D) left atrial (LA) reservoir longitudinal strain, (E) left ventricular ejection fraction (LVEF), (F) left ventricular (LV) mechanical dispersion. ULOA =upper limit of agreement, LLOA =lower limit of agreement

The core laboratory inter‐observer variability in a subset of 30 participants is depicted in Table 4 and Figure 3. Reproducibility of the main outcome measures, LVEF (ICC 0.85) and GLS (ICC 0.76), were good to excellent. The mean values of these measurements ranged from 41% to 66% (LVEF) and −15% to −27% (GLS). No proportional biases were observed for any of the primary outcome measurements (linear regression LVEF *P* =.285; GLS *P* =.131). Proportional biases for diastolic septal thickness and fractional shortening could not be excluded (*P* =.03 and 0.04, respectively), but resolved when deleting two highly influential extreme datapoints. Relative to the mean value, the limits of agreement were wide for LV mechanical dispersion (−53 to 43%), septal and posterior wall thickness (−52 to 35% and −47 to 36%, respectively), left atrial volume (−47 to 34%), and fractional shortening (−29 to 49%).

**TABLE 4:** Inter‐observer variability for the echocardiographic measurements in 30 patients

Measure	Mean	Range	Absolute			Relative (%)^a^			ICC
			Difference ±SD	95% LOA	*p*	Difference ±SD	95% LOA	*p*	
LVEDD (mm)	47.3	35 ‐ 62	‐0.2 ± 3.6	‐7.2 / 6.7	0.71	0 ± 9	‐17 / 16	0.84	0.82
LVESD (mm)	31.9	23 ‐ 52	‐1.9 ± 2.7	‐7.4 / 3.5	0.001	‐6 ± 9	‐25 / 12	0.002	0.87
Septal thickness (mm)	7.4	4 ‐ 11	‐0.7 ± 1.6	‐4.0 / 2.4	0.02	‐9 ± 22	‐52 / 35	0.047	0.51
Posterior wall thickness (mm)	8.8	6 ‐ 15	‐0.4 ± 1.8	‐4.0 / 3.0	0.20	‐6 ± 21	‐47 / 36	0.18	0.61
Left atrial volume (ml)	39.4	21 ‐ 84	‐2.8 ± 8.1	‐18.6 / 13.0	0.09	‐6 ± 21	‐47 / 34	0.15	0.83
E wave (cm/s)	81.1	46 ‐ 152	0.9 ± 4.7	‐8.3 / 10.0	0.33	1 ± 7	‐12 / 14	0.60	0.98
A wave (cm/s)	55.7	38 ‐ 148	0.2 ± 6.8	‐13.2 / 13.6	0.12	3 ± 12	‐20 / 26	0.15	0.94
TDI LV e’ lateral (cm/s)	14.6	6 ‐ 25	0.4 ± 0.8	‐1.2 / 1.9	0.01	3 ± 6	‐8 / 13	0.015	0.98
TAPSE (mm)	21.8	17 ‐ 33	‐2.0 ± 3.1	‐8.1 / 4.1	0.74	‐2 ± 13	‐28 / 24	0.35	0.72
Fractional shortening (%)	33.0	16 ‐ 45	3.7 ± 6.0	‐8.1 / 15.5	0.004	10 ± 20	‐29 / 49	0.02	0.63
LV ejection fraction (%) ^b^	54.9	41 ‐ 66	1.8 ± 3.2	‐4.6 / 8.1	0.008	3 ± 6	‐9 / 16	0.02	0.85
Global longitudinal strain (%)	‐19.6	‐15 ‐ −27	‐0.2 ± 1.7	‐3.6 / 3.1	0.45	1 ± 8	‐16 / 18	0.52	0.76
LV mechanical dispersion (msec)	40.0	27 ‐ 58	‐1.5 ± 8.9	‐18.9 / 15.9	0.39	5 ± 24	‐53 / 43	0.30	0.66
Global circumferential strain (%)	‐20.9	‐17 ‐ −27	‐1.0 ± 1.7	‐4.1 / 2.0	0.000	5 ± 8	‐11 / 20	0.000	0.70
RV free wall strain (%)	‐32.1	‐27 ‐ −41	‐0.1 ± 1.7	‐3.4 / 3.2	0.72	0 ± 5	‐10 / 11	0.73	0.89
LA reservoir strain (%)	44.9	21 ‐ 64	‐0.8 ± 4.9	‐10.5 / 8.9	0.38	‐2 ± 11	‐24 / 19	0.25	0.89

Abbreviations: ICC, intra‐class correlation coefficient; LA, left atrium; LOA, limits of agreement; LV, left ventricle; LVEDD, LV end‐diastolic diameter; LVESD, LV end‐systolic diameter; RV, right ventricle; TAPSE, tricuspid annular plane excursion; TDI, Tissue Doppler Imaging.

^a^ As a percentage of the mean measurement of the two observers

^b^ autoEF n = 24; manual EF n = 4

Intra‐observer analysis (Table 5) generally showed slightly higher ICCs and smaller limits of agreement, as expected. ICCs for LVEF and strain measurements were all ≥0.79, without any proportional biases.

**TABLE 5:** Intra‐observer variability for the echocardiographic measurements in 30 patients

Measure	Mean	Range	Absolute			Relative (%) ^a^			ICC
			Difference ±SD	95% LOA	*p*	Difference ±SD	95% LOA	*p*	
LVEDD (mm)	46.7	38 ‐ 61	‐0.3 ± 2.7	‐5.6 / 5.0	0.55	‐1 ± 6	‐12/ 11	0.47	0.88
LVESD (mm)	31.0	22 ‐ 52	‐0.6 ± 1.9	‐4.3 / 3.1	0.08	‐2 ± 7	‐16 / 11	0.09	0.95
Septal thickness (mm)	7.0	5 ‐ 10	0.3 ± 1.0	‐1.7 / 2.3	0.12	4 ± 13	‐23 / 32	0.10	0.74
Posterior wall thickness (mm)	8.7	7 ‐ 13	0.2 ± 1.8	‐3.4 / 3.7	0.62	2 ± 22	‐42 / 45	0.71	0.56
Left atrial volume (ml)	36.9	17 ‐ 84	‐0.8 ± 6.3	‐13.1 / 11.3	0.49	‐1 ± 18	‐36 / 33	0.71	0.90
E wave (cm/s)	81.5	43 ‐ 149	0.0 ± 3.0	‐5.8 / 5.8	1	‐1 ± 5	‐10 / 9	0.47	0.99
A wave (cm/s)	56.5	36 ‐ 147	0.4 ± 2.7	‐4.9 / 5.7	0.42	1 ± 5	‐10 / 11	0.43	0.99
TDI LV e’ lateral (cm/s)	14.8	6 ‐ 25	0.1 ± 0.7	‐1.3 / 1.5	0.45	1 ± 5	‐9 / 10	0.62	0.99
TAPSE (mm)	21.5	12 ‐ 29	‐0.3 ± 1.6	‐3.4 / 2.8	0.27	‐2 ± 7	‐17 / 12	0.11	0.93
Fractional shortening (%)	34.1	16 ‐ 49	0.8 ± 5.3	‐9.6 / 11.3	0.40	‐2 ± 18	‐33 / 37	0.57	0.79
LV ejection fraction (%) ^b^	55.6	39 ‐ 65	0.3 ± 3.6	‐6.7 / 7.3	0.67	1 ± 7	‐12 / 13	0.65	0.87
Global longitudinal strain (%)	‐19.1	‐14 ‐ −24	0.5 ± 1.2	‐2.0 / 2.9	0.06	‐2 ± 6	‐15 / 10	0.05	0.82
LV mechanical dispersion (msec)	41.3	21 ‐ 59	0.2 ± 5.6	‐10.7 / 11.1	0.83	‐0.2 ± 14	‐28 / 27	0.93	0.86
Global circumferential strain (%)	‐20.2	‐15 ‐ −25	‐0.1 ± 1.5	‐3.1 / 2.9	0.60	1 ± 8	‐15 / 16	0.59	0.82
RV free wall strain (%)	‐31.8	‐25 ‐ −40	0.4 ± 1.9	‐3.3 / 4.1	0.25	‐1 ± 6	‐13 / 11	0.27	0.85
LA reservoir strain (%)	43.3	26 ‐ 54	0.0 ± 4.6	‐8.9 / 9.0	0.96	0 ± 11	‐21 / 21	0.93	0.79

Abbreviations: ICC, intra‐class correlation coefficient; LA, left atrium; LOA, limits of agreement; LV, left ventricle; LVEDD, LV end‐diastolic diameter; LVESD, LV end‐systolic diameter; RV, right ventricle; TAPSE, tricuspid annular plane excursion; TDI, Tissue Doppler Imaging.

^a^ As a percentage of the mean the two measurements.

^b^ autoEF n = 24; manual EF n = 4.

## 4 DISCUSSION

This echocardiography protocol of the Dutch nationwide DCCSS LATER 2 CARD study is unique in its comprehensiveness incorporating well‐established conventional echocardiographic parameters on systolic and diastolic performance, as well as advanced quantification of myocardial function using strain measurements obtained in vendor‐independent software. We show high feasibility and reproducibility of the measurements, which will support the generalizability of the conclusions from our main outcome analyses.

In a comparable cohort from the St. Jude Lifetime Study that included 1,820 adult long‐term CCS, Armstrong et al evaluated cardiotoxicity in CCS during an outpatient clinic visit with echocardiography. This study also provided a very detailed cardiac assessment including LVEF, GLS, and GCS as systolic function parameters, diastolic function indices and exercise capacity.^12^ Our study has the potential to complement existing data, especially for smaller subgroups in the very heterogeneous population of CCS. Furthermore, it will allow for a detailed description of (combinations of) multiple systolic and diastolic function abnormalities in CCS, and combination with electrocardiograms and blood biomarkers. Especially, RV and LA strain are relatively new strain measurements, in addition to the contemporary measurements of GLS.

### 4.1 Feasibility

The feasibility of the (advanced) echocardiographic measurements was higher in sibling controls than in the participating CCS. Although our overall image quality rating was not different between CCS and siblings, it might not be robust enough to detect subtle differences. The feasibility of measurements itself may be regarded as a more objective and detailed manifestation of individual image quality. There may be a number of reasons for this difference. First, image quality might be affected more often in CCS, due to consequences of radiotherapy exposure or surgery on the chest region, and different body composition. Second, siblings were invited in a later stage than CCS, when sonographers and centers may have been better familiarized with the acquisition protocol. Of note, the core laboratory measurements did not start before siblings were included, to ensure blinding.

Third, to prevent missing CCS with less frequent surveillance or with more severe cardiotoxicity, we included some additional echocardiograms as stated in the methods section.

Nevertheless, the feasibility of GLS measurement in 80% in the context of an acquisition protocol is comparable to that of the Normal Reference Ranges for Echocardiography study.^17^

### 4.2 Core laboratory variability

Generally, the inter‐observer variability of conventional and strain measurements in the present study lies within the ranges reported in the literature.^9^, ^12^, ^17^, ^31^ Two measurements are of particular importance to discuss.

First, the reproducibility of biplane LVEF is comparable to that of GLS, which we attribute to the use of the semi‐automated endocardial border tracking option for LVEF measurement. This functionality is based on speckle tracking and minimizes user interaction, which has been shown to reduce inter‐observer variability and the need for expert readers, compared with manual contouring.^32^, ^33^

Second, we chose not to include global radial strain (GRS) in our protocol. GRS has been reported to have inferior reproducibility compared to GCS,^17^, ^31^, ^34^ although it is acquired during the same analysis in the short‐axis view. The GRS measurement is subject to the lower lateral than axial resolution of echocardiographic images. The specific software we use offers a useful function to aid tracking by adjusting the end‐diastolic contours after the myocardium has been tracked. Since these corrections entail both the endocardial and epicardial contours, we judged the pitfall of accumulating manual measurement errors, that are relatively large compared with the myocardial wall thickness, to be unacceptable. Our data support findings in the literature that GCS measurements are more difficult to reproduce than GLS measurements.^17^, ^31^

Notably, LV mechanical dispersion showed acceptable ICCs for inter‐ and intra‐observer variability, but with wide limits of agreement (11‐19msec, 27%‐53%) relative to the measured values. These limits of agreement are accepted in the field, as differences of 20msec were shown relevant for predicting arrhythmias.^28^

### 4.3 Myocardial strain

Myocardial strain imaging has not been fully adopted by the chamber quantification guidelines,^22^ but has been endorsed by the adult cardiotoxicity imaging expert consensus.^11^ As there is sufficient evidence that GLS is feasible, reproducible and adds prognostic value in a variety of cardiovascular diseases,^7^, ^8^, ^15^ inclusion of GLS in guidelines may only be a matter of time. GLS assessment may serve other purposes than only early initiation of heart failure treatment,^35^ such as selecting low‐risk groups.

A major perceived limitation in using strain measurements is the heterogeneity in proposed cutoff values that are provided by the different software vendors. Variability in published strain values may furthermore depend on event timing and the myocardial layer that is analyzed. A dedicated task force is in place to standardize strain measurements,^9^, ^23^ and more pragmatic cutoff values have been suggested.^36^ In our study, we use a vendor‐independent software that can process vendor‐specific (raw) dicom cine loops from any acquisition station. Continuous strain values might better inform on disease severity, and laboratory‐specific validation of normative values or inclusion of a control population are still of great importance at this stage.

We have provided a detailed description of our myocardial strain measurements, including the handling of not‐tracked segments and GLS calculation, which are important for reproducibility and fair comparisons to normative values.^17^, ^37^ We encourage the use of protocols similar to the current one when performing myocardial strain analyses in large cohorts, including transparent description of calculation methods.

### 4.4 Limitations

Although LVEF measured on three‐dimensional echocardiography proved to be more reproducible and thus more suitable than biplane LVEF to detect subtle changes over time,^10^ three‐dimensional echocardiography was not standard of care in all participating centers and thus not included in the protocol. Three‐dimensional LVEF measurements are available for approximately one‐third of the study population. In addition, reproducibility analysis were performed in a random sample that not include patients with very low LVEF. However, in the setting of surveillance echocardiography the reproducibility in “borderline cases” may be of most importance.

## 5 CONCLUSIONS

The echocardiographic substudy of the nationwide cross‐sectional DCCSS LATER 2 CARD study evaluates the prevalences of contemporary systolic and diastolic function parameters in a large cohort of CCS and has a parallelly included sibling cohort for comparison. It includes a protocolized echocardiogram, with feasible and reproducible primary outcome measurements.

## CONFLICT OF INTEREST

None declared.

## AUTHOR CONTRIBUTIONS

LKr, JLo, CR, WT, MvdHei, HvdP, EvDa, EvDu, MvdHeu, AdV, ML, EF, LKa, YP, CdK, GW, LB, AMaa, and AT involved in concept / design. LKr, JLo, WK, AMav, EF, LKa, YP (Dutch Heart Foundation); LKr, EvDu, MvdHeu, and WT (KiKa/ODAS) involved in funding. RM, JLe, and GW involved in data collection and database management. RM, JLe, and LKa involved in statistics and data analysis. RM, LKa, and WK involved in drafting article. All listed authors involved in interpretation, critical revision, and approval of article.

## Supporting information

Figure S1. Feasibility of measurements depicted for different image qualities. Examples of a B‐mode (LVEF = left ventricular ejection fraction), an M‐mode (TAPSE = tricuspid annular systolic plane excursion) and a speckle tracking (GLS = global longitudinal strain) measurement are shown.

## Data Availability

Individual participant data, after de‐identification, may be shared with investigators who would like to collaborate after the main analyses of the LATER CARD study are finished. Applications of intent can be sent to e.a.m.feijen@prinsesmaximacentrum.nl and will be reviewed by the LATER study group.

## References

[1] Gatta G , Botta L , Rossi S , et al Childhood cancer survival in Europe 1999–2007: results of EUROCARE‐5–a population‐based study. Lancet Oncol. 2014;15(1):35‐47. 24314616 10.1016/S1470-2045(13)70548-5

[2] Hudson MM , Ness KK , Gurney JG , et al Clinical ascertainment of health outcomes among adults treated for childhood cancer. JAMA. 2013;309(22):2371‐2381. 23757085 10.1001/jama.2013.6296 PMC3771083

[3] Feijen E , Font‐Gonzalez A , Van der Pal HJH , et al Risk and Temporal Changes of Heart Failure Among 5‐Year Childhood Cancer Survivors: a DCOG‐LATER Study. J Am Heart Assoc. 2019;8(1):e009122. 30595059 10.1161/JAHA.118.009122 PMC6405698

[4] Fidler MM , Reulen RC , Henson K , et al Population‐Based Long‐Term Cardiac‐Specific Mortality Among 34 489 Five‐Year Survivors of Childhood Cancer in Great Britain. Circulation. 2017;135(10):951‐963. 28082386 10.1161/CIRCULATIONAHA.116.024811 PMC5338891

[5] Mulrooney DA , Hyun G , Ness KK , et al Major cardiac events for adult survivors of childhood cancer diagnosed between 1970 and 1999: report from the Childhood Cancer Survivor Study cohort. BMJ. 2020;368:l6794. 31941657 10.1136/bmj.l6794 PMC7190022

[6] Armenian SH , Hudson MM , Mulder RL , et al Recommendations for cardiomyopathy surveillance for survivors of childhood cancer: a report from the International Late Effects of Childhood Cancer Guideline Harmonization Group. Lancet Oncol. 2015;16(3):e123‐e136. 25752563 10.1016/S1470-2045(14)70409-7 PMC4485458

[7] Oikonomou EK , Kokkinidis DG , Kampaktsis PN , et al Assessment of Prognostic Value of Left Ventricular Global Longitudinal Strain for Early Prediction of Chemotherapy‐Induced Cardiotoxicity: A Systematic Review and Meta‐analysis. JAMA Cardiol. 2019;4(10):1007‐1018. 31433450 10.1001/jamacardio.2019.2952 PMC6705141

[8] Kalam K , Otahal P , Marwick TH . Prognostic implications of global LV dysfunction: a systematic review and meta‐analysis of global longitudinal strain and ejection fraction. Heart. 2014;100(21):1673‐1680. 24860005 10.1136/heartjnl-2014-305538

[9] Farsalinos KE , Daraban AM , Unlu S , et al Head‐to‐Head Comparison of Global Longitudinal Strain Measurements among Nine Different Vendors: The EACVI/ASE Inter‐Vendor Comparison Study. J Am Soc Echocardiogr. 2015;28(10):pp. 1171–1181, e1172. 10.1016/j.echo.2015.06.011 26209911

[10] Thavendiranathan P , Grant AD , Negishi T , et al Reproducibility of echocardiographic techniques for sequential assessment of left ventricular ejection fraction and volumes: application to patients undergoing cancer chemotherapy. J Am Coll Cardiol. 2013;61(1):77‐84. 23199515 10.1016/j.jacc.2012.09.035

[11] Plana JC , Galderisi M , Barac A , et al Expert consensus for multimodality imaging evaluation of adult patients during and after cancer therapy: a report from the American Society of Echocardiography and the European Association of Cardiovascular Imaging. Eur Heart J Cardiovasc Imaging. 2014;15(10):1063‐1093. 25239940 10.1093/ehjci/jeu192 PMC4402366

[12] Armstrong GT , Joshi VM , Ness KK , et al Comprehensive Echocardiographic Detection of Treatment‐Related Cardiac Dysfunction in Adult Survivors of Childhood Cancer: Results From the St. Jude Lifetime Cohort Study. J Am Coll Cardiol. 2015;65(23):2511‐2522. 26065990 10.1016/j.jacc.2015.04.013 PMC4539123

[13] Thavendiranathan P, Poulin F, Lim KD, et al: Use of myocardial strain imaging by echocardiography for the early detection of cardiotoxicity in patients during and after cancer chemotherapy: A systematic review. J Am Coll Cardiol. 2014:63(25 PART A):2751‐2768. 10.1016/j.jacc.2014.01.073 24703918

[14] Leerink JM , Verkleij SJ , Feijen EAM , et al Biomarkers to diagnose ventricular dysfunction in childhood cancer survivors: a systematic review. Heart. 2019;105(3):210‐216. 30158136 10.1136/heartjnl-2018-313634

[15] Magne J , Cosyns B , Popescu BA , et al Distribution and Prognostic Significance of Left Ventricular Global Longitudinal Strain in Asymptomatic Significant Aortic Stenosis: An Individual Participant Data Meta‐Analysis. JACC Cardiovasc Imaging. 2019;12(1):84‐92. 30621997 10.1016/j.jcmg.2018.11.005

[16] Mavinkurve‐Groothuis AM , Groot‐Loonen J , Marcus KA , et al Myocardial strain and strain rate in monitoring subclinical heart failure in asymptomatic long‐term survivors of childhood cancer. Ultrasound Med Biol. 2010;36(11):1783‐1791. 20870348 10.1016/j.ultrasmedbio.2010.08.001

[17] Sugimoto T , Dulgheru R , Bernard A , et al Echocardiographic reference ranges for normal left ventricular 2D strain: results from the EACVI NORRE study. Eur Heart J Cardiovasc Imaging. 2017;18(8):833‐840. 28637227 10.1093/ehjci/jex140

[18] Leerink JM , Feijen E , van der Pal HJH , et al Diagnostic tools for early detection of cardiac dysfunction in childhood cancer survivors: Methodological aspects of the Dutch late effects after childhood cancer (LATER) cardiology study. Am Heart J. 2019;219:89‐98. 31733449 10.1016/j.ahj.2019.10.010

[19] Amzulescu MS , De Craene M , Langet H , et al Myocardial strain imaging: review of general principles, validation, and sources of discrepancies. Eur Heart J Cardiovasc Imaging. 2019;20(6):605‐619. 30903139 10.1093/ehjci/jez041 PMC6529912

[20] Rosner A , Barbosa D , Aarsaether E , et al The influence of frame rate on two‐dimensional speckle‐tracking strain measurements: a study on silico‐simulated models and images recorded in patients. Eur Heart J Cardiovasc Imaging. 2015;16(10):1137‐1147. 25762560 10.1093/ehjci/jev058

[21] Nagata Y , Takeuchi M , Mizukoshi K , et al Intervendor variability of two‐dimensional strain using vendor‐specific and vendor‐independent software. J Am Soc Echocardiogr. 2015;28(6):630‐641. 25747915 10.1016/j.echo.2015.01.021

[22] Lang RM , Badano LP , Mor‐Avi V , et al Recommendations for Cardiac Chamber Quantification by Echocardiography in Adults: An Update from the American Society of Echocardiography and the European Association of Cardiovascular Imaging. European Heart Journal ‐ Cardiovascular Imaging. 2015;16(3):233‐271. 25712077 10.1093/ehjci/jev014

[23] Voigt JU , Pedrizzetti G , Lysyansky P , et al Definitions for a common standard for 2D speckle tracking echocardiography: consensus document of the EACVI/ASE/Industry Task Force to standardize deformation imaging. Eur Heart J Cardiovasc Imaging. 2015;16(1):1‐11. 25525063 10.1093/ehjci/jeu184

[24] Badano LP , Kolias TJ , Muraru D , et al Standardization of left atrial, right ventricular, and right atrial deformation imaging using two‐dimensional speckle tracking echocardiography: a consensus document of the EACVI/ASE/Industry Task Force to standardize deformation imaging. Eur Heart J Cardiovasc Imaging. 2018;19(6):591‐600. 29596561 10.1093/ehjci/jey042

[25] Negishi K , Negishi T , Kurosawa K , et al Practical guidance in echocardiographic assessment of global longitudinal strain. JACC Cardiovasc Imaging. 2015;8(4):489‐492. 25129519 10.1016/j.jcmg.2014.06.013

[26] Unlu S , Duchenne J , Mirea O , et al Impact of apical foreshortening on deformation measurements: a report from the EACVI‐ASE Strain Standardization Task Force. Eur Heart J Cardiovasc. Imaging. 2019. 10.1093/ehjci/jez189 31361311

[27] Thavendiranathan P , Negishi T , Cote MA , et al Single Versus Standard Multiview Assessment of Global Longitudinal Strain for the Diagnosis of Cardiotoxicity During Cancer Therapy. JACC Cardiovasc Imaging. 2018;11(8):1109‐1118. 29778856 10.1016/j.jcmg.2018.03.003

[28] Kawakami H , Nerlekar N , Haugaa KH , et al Prediction of Ventricular Arrhythmias With Left Ventricular Mechanical Dispersion: A Systematic Review and Meta‐Analysis. JACC Cardiovasc Imaging. 2020;13(2 Pt 2):562‐572. 31202762 10.1016/j.jcmg.2019.03.025

[29] Nagueh SF , Smiseth OA , Appleton CP , et al Recommendations for the Evaluation of Left Ventricular Diastolic Function by Echocardiography: An Update from the American Society of Echocardiography and the European Association of Cardiovascular Imaging. J Am Soc Echocardiogr. 2016;29(4):277‐314. 27037982 10.1016/j.echo.2016.01.011

[30] Bujang MA . A simplified guide to determination of sample size requirements for estimating the value of intraclass correlation coefficient: A review. Archives of Orofacial Sciences. 2017;12:1‐11.

[31] Kocabay G , Muraru D , Peluso D , et al Normal left ventricular mechanics by two‐dimensional speckle‐tracking echocardiography. Reference values in healthy adults. Rev Esp Cardiol (Engl Ed). 2014;67(8):651‐658. 25037544 10.1016/j.rec.2013.12.009

[32] Franchi F , Cameli M , Taccone FS , et al Assessment of left ventricular ejection fraction in critically ill patients at the time of speckle tracking echocardiography: intensivists in training for echocardiography versus experienced operators. Minerva Anestesiol. 2018;84(11):1270‐1278. 29648414 10.23736/S0375-9393.18.12249-8

[33] Phad N , de Waal K . Left ventricular ejection fraction using manual and semi‐automated biplane method of discs in very preterm infants. Echocardiography. 2020. 10.1111/echo.14784 32618392

[34] Oxborough D , George K , Birch KM . Intraobserver reliability of two‐dimensional ultrasound derived strain imaging in the assessment of the left ventricle, right ventricle, and left atrium of healthy human hearts. Echocardiography. 2012;29(7):793‐802. 22506912 10.1111/j.1540-8175.2012.01698.x

[35] Thavendiranathan P , Negishi T , Somerset E , et al Strain‐Guided Management of Potentially Cardiotoxic Cancer Therapy. J Am Coll Cardiol. 2020. 10.1016/j.jacc.2020.11.020 33220426

[36] Liu JE , Barac A , Thavendiranathan P , et al Strain Imaging in Cardio‐Oncology. JACC: CardioOncology. 2020;2(5):677‐689. 10.1016/j.jaccao.2020.10.011 PMC8352045 34396282

[37] Cheng S , Larson MG , McCabe EL , et al Age‐ and sex‐based reference limits and clinical correlates of myocardial strain and synchrony: the Framingham Heart Study. Circ Cardiovasc Imaging. 2013;6(5):692‐699. 23917618 10.1161/CIRCIMAGING.112.000627 PMC3856433

[38] Baumgartner H , Hung J , Bermejo J , et al Recommendations on the Echocardiographic Assessment of Aortic Valve Stenosis: A Focused Update from the European Association of Cardiovascular Imaging and the American Society of Echocardiography. J Am Soc Echocardiogr. 2017;30(4):372‐392. 28385280 10.1016/j.echo.2017.02.009

[39] Baumgartner H , Hung J , Bermejo J , et al Echocardiographic assessment of valve stenosis: EAE/ASE recommendations for clinical practice. J Am Soc Echocardiogr. 2009;22(1):pp. 1–23; quiz 101–102. 10.1016/j.echo.2008.11.029 19130998

[40] Lancellotti P , Tribouilloy C , Hagendorff A , et al Recommendations for the echocardiographic assessment of native valvular regurgitation: an executive summary from the European Association of Cardiovascular Imaging. Eur Heart J Cardiovasc Imaging. 2013;14(7):611‐644. 23733442 10.1093/ehjci/jet105

