# Supplementary material for: Echocardiography protocol for early detection of cardiac dysfunction in childhood cancer survivors in the multicenter DCCSS LATER 2 CARD study: Design, feasibility, and reproducibility
Source: Echocardiography. 2021 May 20;38(6):951–63. doi: 10.1111/echo.15081 (PMC8251836; doi:10.1111/echo.15081)
Supplement: Supplementary file 1 — Figure S1. Feasibility of measurements depicted for different image qualities. Examples of a B‐mode (LVEF = left ventricular ejection fraction), an M‐mode (TAPSE = tricuspid annular systolic plane excursion) and a speckle tracking (GLS = global longitudinal strain) measurement are shown. [file ECHO-38-951-s001.pdf]

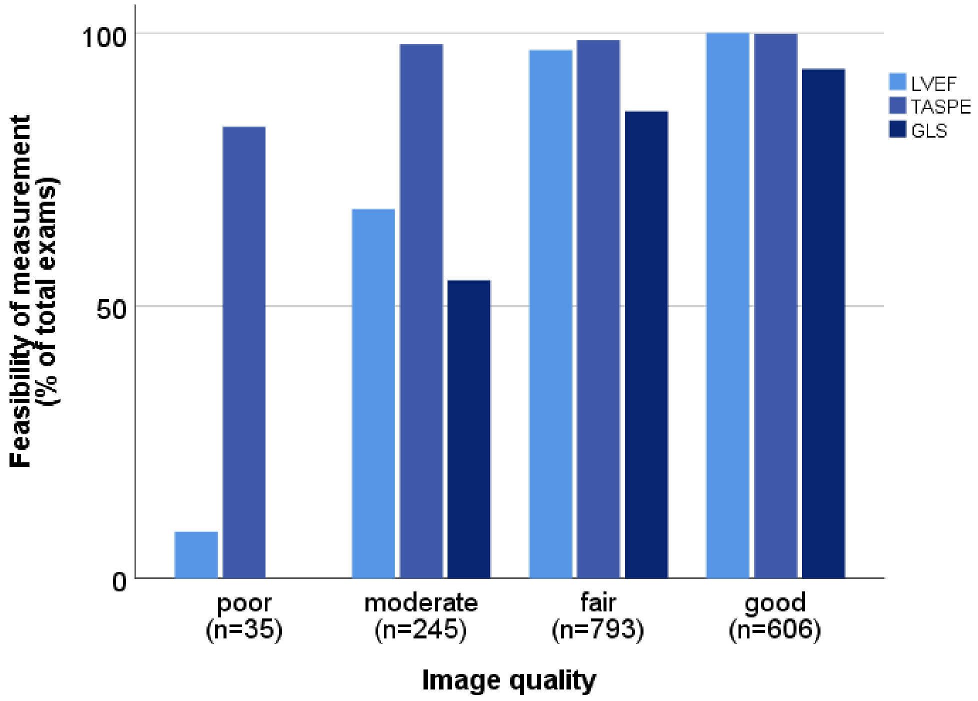

SUPPLEMENTAL FIGURE 1. Feasibility of measurements depicted for different image qualities. Examples of a B-mode (LVEF = left ventricular ejection fraction), an M-mode (TAPSE = tricuspid annular systolic plane excursion) and a speckle tracking (GLS = global longitudinal strain) measurement are shown.
